# The U-shaped effect of coach-athlete attachment on athlete engagement: the mediating role of thriving and the moderating role of mental toughness

**DOI:** 10.3389/fpsyg.2025.1544860

**Published:** 2025-03-07

**Authors:** Zhidong Dai, Tianchen Zheng, Rongzhi Li

**Affiliations:** ^1^School of Physical Education, Shanghai University of Sport, Shanghai, China; ^2^School of Physical Education, Chuzhou University, Chuzhou, Anhui, China

**Keywords:** coach-athlete attachment, thriving, athlete engagement, mental toughness, U-shaped relationship

## Abstract

Based on the Conservation of Resources Theory and Affective Information Theory, this study explored the impact of coach-athlete attachment on athlete engagement, its underlying mechanisms, and boundary conditions from a “loss-gain” dual-path perspective. Using the Coach-Athlete Attachment Scale, Thriving Scale, Athlete Engagement Scale, and Mental Toughness Scale, a cross-sectional survey was conducted with 424 athletes (299 males, 125 females, mean age = 16.14 ± 2.24 years) from different regions, using a convenience sampling method. The results showed that coach-athlete attachment and its subdimensions (avoidant attachment and anxious attachment) exerted a U-shaped influence on thriving and athlete engagement, with an asymmetric U-shaped curve, where the left path is longer and the right path was shorter. Thriving significantly positively influenced athlete engagement and serves as an instantaneous mediator in the U-shaped relationship between coach-athlete attachment and athlete engagement. Mental toughness significantly moderated the U-shaped effect of coach-athlete attachment on thriving and athlete engagement. The findings encouraged coaches to thoughtfully consider athletes’ attachment tendencies and adjust their communication strategies based on athletes’ attachment types to enhance athletes’ thriving and engagement levels.

## Introduction

In competitive sports, athlete engagement is a key predictor of training effectiveness, career sustainability, and competition success (Curran et al., 2015; Lonsdale et al., 2007; Wei et al., 2013). Research shows that athlete engagement is shaped by both endogenous factors (e.g., self-motivation, mental toughness) and exogenous factors (e.g., coach-athlete relationships, leadership behaviors) (De Francisco et al., 2018; Gao et al., 2021; Gu and Xue, 2022; Ye et al., 2016). While endogenous factors act as proximal antecedents, they are influenced by distal external factors, including the dynamic coach-athlete relationship. Coaches’ impact on engagement is mediated through close interaction and communication with athletes. Despite this, existing studies often overlook the bidirectional nature of these relationships, especially the emotional connection (e.g., coach-athlete attachment) and its influence on engagement (Ye et al., 2016).

Coach-athlete attachment can be divided into the “insecure” attachment styles of avoidant attachment and anxious attachment (Ascone et al., 2020). For a long time, the academic focus has been on their negative effects on individuals, such as issues with attention (Pallini et al., 2019), emotion regulation (Liu and Ma, 2019), and interpersonal relationships (Zhang et al., 2023). However, the overwhelming emphasis on these negative outcomes has led to the neglect of some potential benefits of coach-athlete attachment. Recent research has highlighted that both anxious attachment and avoidant attachment can have positive effects on individual behavior. For instance, individuals with anxious attachment expect care and are therefore more proactive in seeking support (Yang, 2018). Athletes with avoidant attachment are less likely to experience conflicts with their coaches (Davis and Jowett, 2014). This suggests that “insecure attachment” may also contain certain “secure” components. The prevailing deficit-focused lens risks pathologizing athletes with insecure attachment, potentially neglecting intervention opportunities that leverage their unique relational strategies. This study aims to construct a theoretical model to explore the potential curvilinear relationship between coach-athlete attachment and athlete engagement, as well as the underlying mechanisms. The findings may contribute to the development of effective coaching strategies and enhance athlete engagement levels.

### Literature review and hypothesis development

Avoidant attachment and anxious attachment in the coach-athlete relationship are typically considered forms of insecure attachment. Athletes with anxious attachment often worry about being rejected or abandoned by their coaches, making it difficult for them to maintain harmonious relationships. These athletes are less positively influenced and more negatively affected by such interactions. On the other hand, athletes with avoidant attachment invest less emotionally in their interactions with coaches, maintaining a certain mental and emotional distance, and perceiving the coach-athlete relationship as less significant (Felton and Jowett, 2013a). Both avoidant and anxious attachment negatively impact the development of a high-quality coach-athlete relationship, which is a critical factor for promoting thriving and athlete engagement (Gu et al., 2023). Moreover, evidence suggests that individuals with anxious attachment allocate significant attentional resources to attachment-related information, while those with avoidant attachment suppress such information, leading to impaired working memory and attentional difficulties (Ma et al., 2016). Impairments in working memory and attentional difficulties are likely to negatively affect athletes’ training and learning, subsequently reducing their levels of thriving and engagement. These challenges could push athletes into a spiral of resource depletion (Hobfoll et al., 2018).

However, evidence suggests that anxious attachment and avoidant attachment also have positive behavioral pathways. Both are conceptualizations of individual emotions, and according to the affect-as-information theory, emotions carry informational functions. Negative emotions can act as warning signals, motivating individuals to put in more effort to acquire resources and overcome difficulties (Schwarz and Clore, 1983). For instance, anxiety can foster proactive problem-solving motivation and positive cognition (Mao et al., 2021), prompting individuals to exert greater effort to gain support. Although individuals with anxious attachment fear rejection or abandonment in relationships, they are more likely to perceive minor incidents as threats and reflect on them, leading to efforts to seek support (Albert et al., 2015). As a result, individuals with anxious attachment, driven by their desire for care, tend to actively seek assistance (Yang, 2018). Avoidance is also a manifestation of negative emotions, but the interpersonal avoidance behavior it triggers can contribute to resource conservation and stress relief (Wei et al., 2022). Moreover, athletes with avoidant attachment are less likely to experience conflicts with their coaches (Davis and Jowett, 2014), and they tend to be more sensitive to supportive behaviors from coaches (Felton and Jowett, 2013b). This responsiveness can enhance their ability to manage stress and maintain focus. From the perspective of COR theory, both anxious and avoidant attachment styles can trigger resource preservation or acquisition behaviors. These behaviors—such as seeking support, self-directed learning, and effortful problem-solving—help athletes either replenish or conserve their resources, thereby promoting thriving and engagement. In this way, athletes may enter a resource gain spiral (Hobfoll et al., 2018).

Including mediating factors in relational pathways allows for a more comprehensive understanding of the influence process between variables (Bollen, 1989). Therefore, it is essential to elucidate the process mechanism through which coach-athlete attachment affects athlete engagement. Thriving is a positive psychological state characterized by both vitality and learning. It enables athletes to engage in training and competition with higher enthusiasm and optimal performance, ultimately enhancing their engagement and achievements (Wang, 2018). As a measure of individual progress and growth (Schaufeli et al., 2006), thriving is commonly associated with numerous positive variables. For example, thriving positively influences athlete engagement (Gu et al., 2023).

The influence of coach-athlete attachment on athlete engagement may vary across individuals. Exploring whether this influence is moderated by personal traits can help coaches tailor their strategies to athletes with different characteristics. Mental toughness, as a critical personal trait, is particularly important for athletes frequently exposed to challenges and pressures. It not only determines whether athletes can persist in their goals under competitive pressure or stress (Crampton, 2015), but also represents a positive psychological resource for coping with adverse circumstances (Rutter, 2006). The Conservation of Resources Theory posits that individuals’ resource reserves are closely related to their likelihood of experiencing resource loss and their toughness to such loss (Hobfoll, 2001). Individuals with abundant initial resources are less likely to lose resources and more capable of acquiring new ones. Conversely, those with fewer initial resources are more susceptible to resource loss and face greater challenges in acquiring additional resources. High-toughness individuals possess greater initial resources, enabling them to better prevent resource depletion and facilitate positive outcomes (Mäkikangas et al., 2010). On one hand, athletes with high mental toughness exhibit higher self-efficacy and coping effectiveness (Nicholls et al., 2011; Kaiseler et al., 2009). These initial resources reduce their psychological discomfort caused by avoidant behavior or poor relationships with coaches and enable them to better manage the negative emotions and stress associated with anxious attachment. Consequently, the negative effects of coach-athlete attachment on thriving and athlete engagement are diminished. On the other hand, high-toughness athletes have stronger control beliefs regarding stressors and superior emotional regulation abilities (Yang et al., 2017). These resource reserves enhance their cognitive flexibility, foster mutually beneficial behaviors with coaches, and help them perceive stress as a challenge rather than a threat. This mindset encourages athletes to participate in training with greater positivity and vitality, thereby strengthening the positive effects of coach-athlete attachment on thriving and athlete engagement (Guo et al., 2021; Reschly et al., 2008).

**Figure 1:**
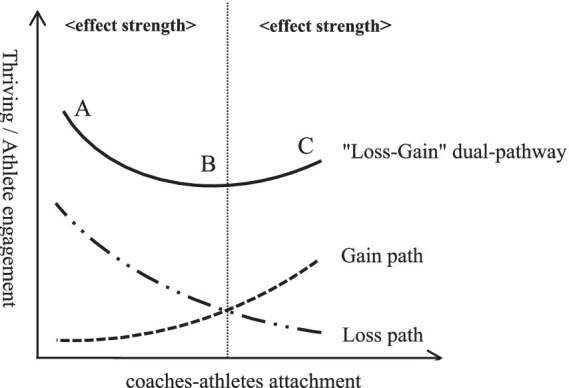
Dual path analysis of the impact of coaches-athletes attachment on thriving and athlete engagement.

In conclusion, the influence of coach-athlete attachment on thriving and athlete engagement is not unidirectional. Exploring this relationship from a dual-path perspective of “depletion-gain” may offer more explanatory power. Under the combined effects of these two pathways, a new composite trajectory emerges. Specifically, when coach-athlete attachment exceeds a certain threshold, the composite trajectory reverses, forming a U-shaped curve (Figure 1). Additionally, mental toughness can moderate this composite path. As mental toughness increases, the positive effects of coach-athlete attachment on thriving and athlete engagement are amplified, while the depletion effects are weakened. Accordingly, the following hypotheses are proposed:

*H1*: There is a U-shaped relationship between coach-athlete attachment (anxious/avoidant) and thriving, as well as athlete engagement, and this U-shaped relationship can be mediated by thriving.

*H2*: Mental toughness significantly moderates the U-shaped relationship between coach-athlete attachment and thriving, as well as athlete engagement.

**Figure 2:**
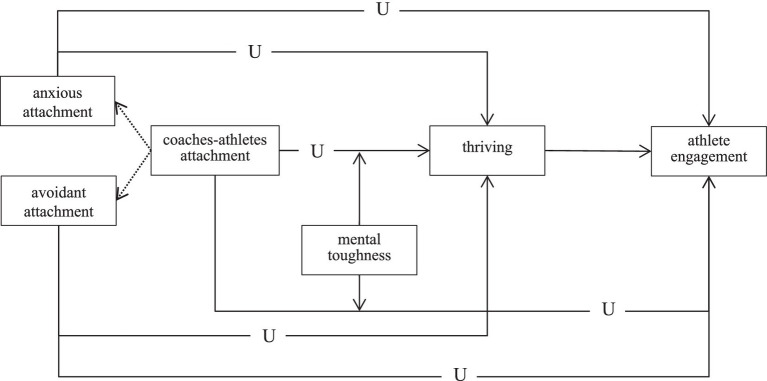
Hypothesis model of the U-shaped relationship between coach-athlete attachment and athlete engagement.

To visually present the relationships among the variables in this study, a theoretical model is constructed as shown in Figure 2.

## Method

### Participants

To ensure the generalizability of the research findings, two inclusion criteria were established: (1) athletes registered at the municipal or provincial level, and (2) athletes participating in ball sports. For reliability, two exclusion criteria were also set: (1) athletes who had been out of training for more than 4 weeks due to injury or other reasons, and (2) athletes with less than 2 years of coach-athlete mentorship. According to statistical theory, the sample size for a questionnaire survey should be 5–10 times the number of scale items (Stuart and Ord, 2010). Therefore, the sample size should have ranged from 240 to 480 participants. A total of 509 questionnaires were distributed through convenience sampling. After screening for invalid responses based on criteria such as completion time less than 5 min, reverse question checks, and consistent answering patterns, 85 invalid questionnaires were removed. Finally, 424 valid questionnaires were obtained, resulting in an effective response rate of 83%. The average age of the athletes was 16.14 years (SD ± 2.24). Detailed demographic information is provided in Table 1.

**Table 1:** Structural composition of test athletes.

Demographic variables	Details	Number	Percentage/%
Sports events	Table tennis	149	35.1
	Badminton	98	23.1
	Football	60	14.2
	Handball	56	13.2
	Volleyball	38	9
	Basketball	23	5.4
Sports level	National level II Athletes	267	63
	National level athletes	78	18.4
	Elite athlete national	59	13.9
	Elite athletes international	20	4.7
Sex	Male	299	70.5
	Female	125	29.5

### Procedures

Upon approval from the authors’ institutional research ethics board (CZSC2024-020). Participants were recruited from sports schools and training centers in Zhejiang, Jiangsu, Fujian, Guangdong, Anhui, Jiangxi, and Shanghai. Prior to the assessment, informed consent was obtained from all athletes to ensure they fully understood the purpose of the study and their rights as participants. During the questionnaire survey, athletes were provided with detailed instructions, including an introduction to the study’s background, the significance of their participation, and clear guidance on how to respond to the questions. The surveys were conducted either before or after athletes’ training sessions to minimize interference with their routines. The questionnaire was distributed online through the “Wenjuanxing” mini-program in WeChat, allowing participants to complete the survey conveniently using their personal mobile devices. The data collection process ensured anonymity and confidentiality to protect the participants’ privacy.

### Measures

All the scales used in this study were derived from validated scales translated and revised by Chinese scholars. These scales were tested for applicability in Chinese athlete populations and demonstrated good reliability and validity. The questionnaire consisted of five sections: demographic information of the participants, the Coach-Athlete Attachment Scale, the Thriving Scale, the Athlete Engagement Scale, and the Mental Toughness Scale. All scales adopted a 7-point Likert scoring system, ranging from 1 (“strongly disagree”) to 7 (“strongly agree”), with the final score for each scale represented by the average of all item scores.

#### Coach-athlete attachment

The study employed the Coach-Athlete Attachment Scale-Fourteen Items (CAAS-14) developed by Davis and Jowett (2013) and revised by Guo (2016) for use with Chinese populations. This scale consists of three dimensions: secure attachment, anxious attachment, and avoidant attachment. For this study, only the latter two dimensions were selected, comprising a total of 10 items. The Cronbach’s *α* coefficient for the entire scale was 0.895, with 0.867 and 0.905 for anxious attachment and avoidant attachment, respectively. Results from the confirmatory factor analysis (CFA) indicated good model fit: χ^2^/df = 2.86, RMSEA = 0.06, SRMR = 0.02, CFI = 0.96, TLI = 0.97.

#### Thrivin

The study used the Athlete Thriving Scale, developed by Porath et al. (2012) and revised by Ma (2019), which is divided into two dimensions: vitality and learning, comprising a total of 10 items. In this study, the Cronbach’s *α* coefficient for the entire scale was 0.889, with the vitality and learning dimensions showing Cronbach’s α coefficients of 0.84 and 0.82, respectively. Confirmatory factor analysis (CFA) demonstrated good model fit: χ^2^/df = 2.39, RMSEA = 0.06, SRMR = 0.03, CFI = 0.98, TLI = 0.97.

#### Athlete engagement

The study utilized the Athlete Engagement Scale, developed by Lonsdale et al. (2007) and revised by Wang Bin et al. This scale consists of four dimensions: confidence, dedication, vitality, and enthusiasm, with a total of 16 items. In this study, the Cronbach’s α coefficient for the entire scale was 0.814. The Cronbach’s α coefficients for confidence, dedication, vitality, and enthusiasm were 0.74, 0.812, 0.789, and 0.85, respectively. The confirmatory factor analysis (CFA) results indicated good model fit: χ^2^/df = 2.86, RMSEA = 0.06, SRMR = 0.05, CFI = 0.90, TLI = 0.91.

#### Mental toughness

The Mental Toughness Scale for athletes used in this study was developed by Sheard et al. (2009) and revised by Bin et al. (2014). This scale comprises three dimensions: perseverance, confidence, and control, with a total of 12 items. In the present study, the Cronbach’s α coefficient for the overall scale was 0.881, while the coefficients for perseverance, confidence, and control were 0.91, 0.838, and 0.872, respectively. Confirmatory factor analysis (CFA) results showed: χ^2^/df = 2.90, RMSEA = 0.07, SRMR = 0.03, CFI = 0.93, and TLI = 0.90.

### Data analysis

The data were processed using SPSS 26 and AMOS 26. First, a common method bias test and reliability and validity tests were conducted on the questionnaire. Next, descriptive analysis was performed to obtain the mean and standard deviation of each variable. Simultaneously, the athlete’s gender, age, education level, competition level, and sports specialization, as well as the coach’s gender and years of coaching experience, were used as control variables in a partial correlation analysis to determine the correlation coefficients among all variables. Finally, a hierarchical regression analysis was conducted to test the research hypotheses. Taking thriving and sport engagement as the dependent variables, hypotheses were tested stepwise by entering variables into the model in the following order: control variables (athletes’ gender, age, education level, competition level, and sports specialization; coaches’ gender and years of experience), independent variables (coach-athlete attachment, anxious attachment, avoidant attachment) and their squared terms, and the interaction terms between the independent variables (and their squared terms) and the moderator variable (mental toughness). This approach was used to examine hypotheses H1 to H2.To reduce multicollinearity and minimize research error, variables involving interaction terms and squared terms were mean-centered before regression analysis. Detailed results are shown in Tables 2, 3. Additionally, Origin 2021 was used to draw three-dimensional response surface plots related to the moderating effects.

**Table 2:** Hierarchical regression analysis results with thriving as the dependent variable.

Variables	Thriving							
	M1	M2	M3	M4	M5	M6	M7	M8
Sex	−0.028	−0.035	0.001	0.022	−0.033	−0.005	0.003	−0.019
Age	−0.083^*^	−0.086^*^	−0.066^*^	−0.081^*^	−0.072^*^	−0.075^*^	−0.029	−0.033
Educational level	0.091	0.162	0.092	0.17	0.087	0.145	0.042	0.057
Sports speciality	0.090^*^	0.093^*^	0.099^**^	0.084^*^	0.088^*^	0.085^*^	0.063^*^	0.064^*^
Sports level	−0.219^*^	−0.221^*^	−0.22^***^	−0.22^***^	−0.22^***^	−0.224^***^	−0.114^***^	−0.114^**^
Coach gender	0.179	0.172	0.179	0.199	0.174	0.182	0.166	0.177
Coach teaching age	−0.198^**^	−0.195^**^	−0.208^**^	−0.198^**^	−0.212^***^	−0.216^***^	−0.173^***^	−0.16^**^
Coach athlete attachment					−0.131^***^	−0.129^***^	−0.523^***^	−0.106^***^
Coach athlete attachment^2^						0.066^***^	0.056^***^	0.058^***^
Mental toughness							0.572^***^	1.236^***^
Coach athlete attachment×Mental toughness								0.257^**^
Coach athlete attachment^2^ × Mental toughness								−0.042^**^
Anxious attachment	−0.098^***^	−0.122^***^						
Anxious attachment^2^		0.074^***^						
Avoidant attachment			−0.091^**^	−0.082^***^				
Avoidant attachment^2^				0.062^***^				
*R* ^2^	0.146	0.195	0.139	0.176	0.153	0.185	0.472	0.491
△R^2^	–	0.049	–	0.037	–	0.032	0.287	0.019
*F*	8.871^***^	11.176^***^	8.403^***^	9.837^***^	9.404^***^	10.475^***^	36.945^***^	33.088^***^
Constant	7.648	7.298	7.281	7.155	7.489	7.228	6.379	6.398

*p < 0.05, **p < 0.01, ***p < 0.001. The coefficients in the table are all unstandardized regression coefficients; △R^2^ refers to the change in R-squared. Superscript value 2 refers to the squaredterm of the variable.

**Table 3:** Hierarchical regression analysis results with athlete engagement as the dependent variable.

Variables	Athlete engagement									
	M9	M10	M11	M12	M13	M14	M15	M16	M17	M18
Sex	0.057	0.046	0.095	0.126	0.054	0.095	0.098	0.099	0.083	0.112
Age	−0.087^*^	−0.093^**^	−0.069^**^	−0.09^**^	−0.075^*^	−0.079^*^	−0.033	−0.054	−0.056	−0.031
Grade	0.035	0.155	0.036	0.153	0.03	0.115	0.026	0.059	0.066	−0.019
Sports speciality	0.075^*^	0.081	0.088^*^	0.066^*^	0.075^*^	0.07^*^	0.018	0.058	0.058	0.027
Sports level	−0.114	−0.116^*^	−0.115^*^	−0.114^*^	−0.115^*^	−0.12^*^	0.017	−0.061	−0.063	0.033
Coach gender	0.153	0.14	0.155	0.183	0.147	0.159	0.047	0.15	0.159	0.041
Coach age	−0.069	−0.064	−0.079	−0.064	−0.085	−0.091	0.042	−0.067	−0.053	0.073
Anxious attachment	−0.118^***^	−0.158^***^								
Anxious attachment^2^		0.127^***^								
Avoidant attachment			−0.103^***^	−0.089^***^						
Avoidant attachment^2^				0.093^***^						
Coach athlete attachment					−0.153^***^	−0.150^***^	−0.482^***^	−0.81^***^	−0.137^***^	
Coach athlete attachment^2^						0.096^***^	0.056^***^	0.091^***^	0.092^***^	
Thriving							0.614^***^			0.669^***^
Mental toughness								0.31^***^	0.752^**^	
Coach athlete attachment×Mental toughness									0.157	
Coach athlete attachment^2^ ×Mental toughness									−0.028^*^	
*R* ^2^	0.121	0.280	0.106	0.177	0.129	0.204	0.540	0.296	0.309	0.505
△*R*^2^	–	0.159	–	0.089	–	0.075	0.336	0.092	0.012	–
*F*	7.141^***^	17.885^***^	6.119^***^	11.086^***^	7.697^***^	11.78^***^	48.466^***^	17.391^***^	15.302^***^	52.82^***^
Constant	7.555	6.955	7.121	6.934	7.360	6.978	2.544	6.518	6.515	2.295

*p < 0.05, **p < 0.01, ***p < 0.001. The coefficients in the table are all unstandardized regression coefficients; △R^2^ refers to the change in R-squared. Superscript value 2 refers to the squaredterm of the variable.

## Results

### Common method bias testing

The Harman’s single-factor test revealed that a total of 11 factors had eigenvalues greater than 1, with the first factor explaining 32.58% of the variance, which was below the critical threshold of 40%. This indicated that there was no significant common method bias in this study.

### Correlation analyses

The results of the correlation analysis were shown in Table 4. There is a significant negative correlation between coach-athlete attachment and both thriving (*r* = −0.216, *p* < 0.001) and athlete engagement (*r* = −0.257, *p* < 0.001). Thriving was positively correlated with both athlete engagement (*r* = 0.688, *p* < 0.001) and mental toughness (*r* = 0.595, *p* < 0.001). Athlete engagement was also positively correlated with mental toughness (*r* = 0.353, *p* < 0.001). However, the correlation between coach-athlete attachment and mental toughness was not significant (*p* > 0.05). The correlation coefficients between the main variables are all less than 0.7, indicating that there is no multicollinearity issue, making the data suitable for regression analysis.

**Table 4:** Partial correlation analysis of coach-athlete attachment, thriving, athlete engagement, and mental toughness.

variables	Coach athlete attachment	Anxious attachment	Avoidant attachment	Thriving	Athlete engagement	Mental toughness	M ± SD
Coach athlete attachment	1						3.684 ± 1.547
Anxious attachment	0.857^***^	1					3.438 ± 1.861
Avoidant attachment	0.844^***^	0.447^***^	1				3.930 ± 1.758
Thriving	−0.216^***^	−0.187^***^	−0.181^***^	1			5.845 ± 0.985
Athlete engagement	−0.257^***^	−0.23^***^	−0.207^***^	0.688^***^	1		6.011 ± 0.940
Mental toughness	−0.065	−0.072	−0.039	0.595^***^	0.353^***^	1	6.138 ± 0.952

***p < 0.001.

### U-type relationship

According to Haans et al. (2016), three conditions must be met to confirm a U-shaped relationship. First, the coefficient of the squared term of the independent variable must be significantly positive. Second, when the independent variable takes its minimum value, the slope of the curve should be negative, and when the independent variable takes its maximum value, the slope should be positive. Third, the value of the U-shaped curve’s extreme point must fall within the range of values for the independent variable.

First, to test the U-shaped relationship with thriving as the dependent variable, based on the results from Model M2, M4, and M6 in Table 2, the coefficients of the squared terms for coach-athlete attachment, anxious attachment, and avoidant attachment are all positive and significant, fulfilling the first condition. The regression equation for the relationship between X (coach-athlete attachment, anxious attachment, avoidant attachment) and Y (thriving) is: Y = *β*_0_ + *β*_1_X + *β*_2_X^2^, The slope equation is:S = 2*β*_2_X + *β*_1_, After centering, the standard values of X range from −2.684 to 3.316. The regression coefficients for Models M2, M4, and M6 were as follows: (*β*_1_ = −0.122, *β*_2_ = 0.074), (*β*_1_ = −0.082, *β*_2_ = 0.062), (*β*_1_ = −0.129, *β*_2_ = 0.066), When X takes its minimum value, the slope values (S) for Models M2, M4, and M6 are: −0.519, −0.415, and − 0.483, respectively, all showing a negative slope. When X takes its maximum value, the S values are: 0.369, 0.329, and 0.309, all showing a positive slope, thereby satisfying the second condition. The extreme point of the U-shaped curve was calculated using the formula X = −*β*_1_/(2*β*_2_). The extreme points for Models M2, M4, and M6 are 0.824, 0.661, and 0.977, respectively, all of which fall within the range of X (−2.684 to 3.316), satisfying the third condition. This indicates that coach-athlete attachment, as well as its sub-dimensions (anxious and avoidant attachment), exert a U-shaped effect on thriving.

Second, to test the U-shaped relationship with athlete engagement as the dependent variable, based on the results from Models M10, M12, and M14 in Table 3, the coefficients of the squared terms for coach-athlete attachment and its sub-dimensions are significantly positive, fulfilling the first condition. The regression coefficients for Models M10, M12, and M14 were: (*β*_1_ = −0.158, *β*_2_ = 0.127), (*β*_1_ = −0.089, *β*_2_ = 0.093), (*β*_1_ = −0.150, *β*_2_ = 0.096). When X takes its minimum value, the slope values (S) for Models M10, M12, and M14 are: −0.840, −0.588, and − 0.665, respectively. When X takes its maximum value, the S values are: 0.684, 0.528, and 0.487, fulfilling the second condition. The extreme points for Models M10, M12, and M14 are 0.662, 0.470, and 0.781, respectively, all falling within the range of X (−2.684 to 3.316), fulfilling the third condition. This indicates that coach-athlete attachment, as well as its sub-dimensions, exerted a U-shaped effect on athlete engagement.

Finally, to determine the specific shape of the U-shaped curves, i.e., the length of the paths on both sides of the curve, we observe that based on the standard value range of X (−2.684 to 3.316), and the extreme point values of each model, all U-shaped curves have their turning points located in the middle to later part of the standard value range. This suggests that the paths on the left side of the curve are longer, and the paths on the right side are shorter, exhibiting an asymmetric U-shape.

### Mediation effect

Model M18 indicates that thriving has a significant positive effect on athlete engagement (*β* = 0.669, *p*<0.001). In Model M15, after including thriving as an independent variable, its coefficient remains significantly positive (*β* = 0.614, *p*<0.001), and the coefficient for the squared term of coach-athlete attachment is still significant (*β* = 0.056, *p*<0.001). This suggests that thriving serves as a partial mediator.

Hierarchical regression analysis revealed a nonlinear relationship between coach-athlete attachment, thriving, and athlete engagement. To test the instantaneous mediating effect of thriving, Hayes’s approach was employed, using the MEDCURVE plug-in for analysis (Hayes and Preacher, 2010). The results, presented in Table 5, indicated that when the values of coach-athlete attachment were set at the mean and ± 1 standard deviation, the confidence intervals do not include zero, demonstrating that the instantaneous mediating effect of thriving is significant. This further confirms that regardless of whether the values of coach-athlete attachment are low, medium, or high, they can indirectly influence athlete engagement through thriving.

**Table 5:** Test results of the instantaneous mediation effect of thriving.

Mediating variables	Values of independent variable	Bootstrap sample size	Confidence intervals		Instantaneous mediation effect
			Upper level	Low level	
Coach athlete attachment-1 standard deviations	2.1373	5,000	−0.131	−0.298	−0.212
Coach athlete attachment mean	3.6842	5,000	−0.041	−0.115	−0.077
Coach athlete attachment+1standard deviations	5.2311	5,000	0.120	0.002	0.057

### Moderated effects

According to the method proposed by Haans et al. (2016), the moderating effect of mental toughness (Z) on the U-shaped relationship between coach-athlete attachment (X) and thriving/athlete engagement (Y) was tested in two steps using the equation Y = *β*_0_ + *β*_1_X + *β*_2_X^2^ + *β*_3_XZ + *β*_4_X^2^Z + *β*_5_Z. In the first step, the direction of the U-shaped curve’s inflection point shift is examined. By setting the first derivative of X to zero, the inflection point is derived as: X^*^ = (−*β*_1_-*β*_3_Z)/(2*β*_2_ + 2*β*_4_Z). The inflection point depended on the moderator variable. Taking the derivative of X* with respect to Z: ∂X^*^/∂Z = (*β*_1_*β*_4_-*β*_2_*β*_3_)/2(*β*_2_ + *β*_4_Z)^2^. If ∂X^*^/∂Z>0, the inflection point of the U-shaped curve shifts to the right as Z increases. Conversely, if ∂X^*^/∂Z<0, the inflection point shifts to the left as Z decreases. In the second step, the steepness of the U-shaped curve was determined. If *β*_4_ is significantly less than zero, the U-shaped curve becomes flatter; if *β*_4_ is significantly greater than zero, the U-shaped curve becomes steeper.

First, the changes in the U-shaped curve between coach-athlete attachment and thriving with increased mental toughness are examined. Based on the results of Model M8, ∂X^*^/∂Z = −141.027, indicating that as mental toughness increased, the inflection point of the U-shaped curve between coach-athlete attachment and thriving shifted to the left, meaning the beneficial effect of coach-athlete attachment on thriving occurred earlier. In the second step, the interaction term between the squared term of coach-athlete attachment and mental toughness is significant (*β*_4_ = −0.042, *p*<0.01), with a negative coefficient, suggesting that as mental toughness increases, the U-shaped curve becomes flatter. In conclusion, mental toughness significantly moderated the U-shaped relationship between coach-athlete attachment and thriving.

Next, the changes in the U-shaped curve between coach-athlete attachment and athlete engagement with increased mental toughness are examined using the same steps. According to Model M17, ∂X^*^/∂Z = −1.054, indicating that as mental toughness increases, the inflection point of the U-shaped curve between coach-athlete attachment and athlete engagement shifts to the left. Model M17 shows that the interaction term between the squared term of coach-athlete attachment and mental toughness is significant (*β*_4_ = −0.028, *p*<0.05), with a negative coefficient, indicating that as mental toughness increases, the U-shaped curve becomes flatter. In conclusion, mental toughness significantly moderates the U-shaped relationship between coach-athlete attachment and athlete engagement.

**Figure 3:**
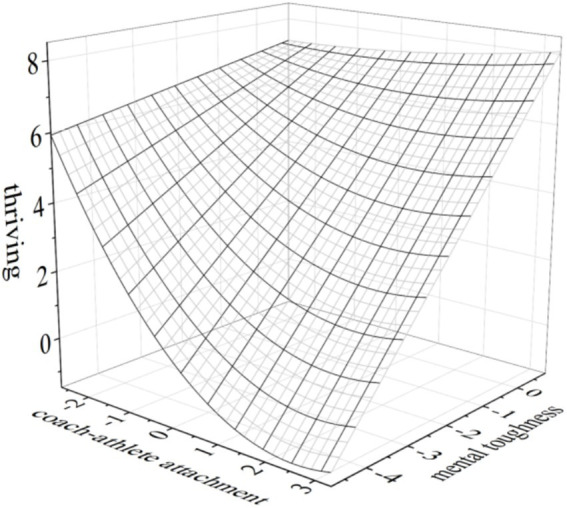
The moderating effect of mental toughness on the U-shaped relationship between coach athlete attachment and thriving. Y (X, Z) = 6.398–0.106X + 0.058×^2^ + 0.257XY-0.042X^2^Y + 1.236Y.Coach athlete attachment∈[−2.684, 3.316]; thriving∈[−1.5, 8.5]; mental toughness ∈[−4.80, 0.86].

**Figure 4:**
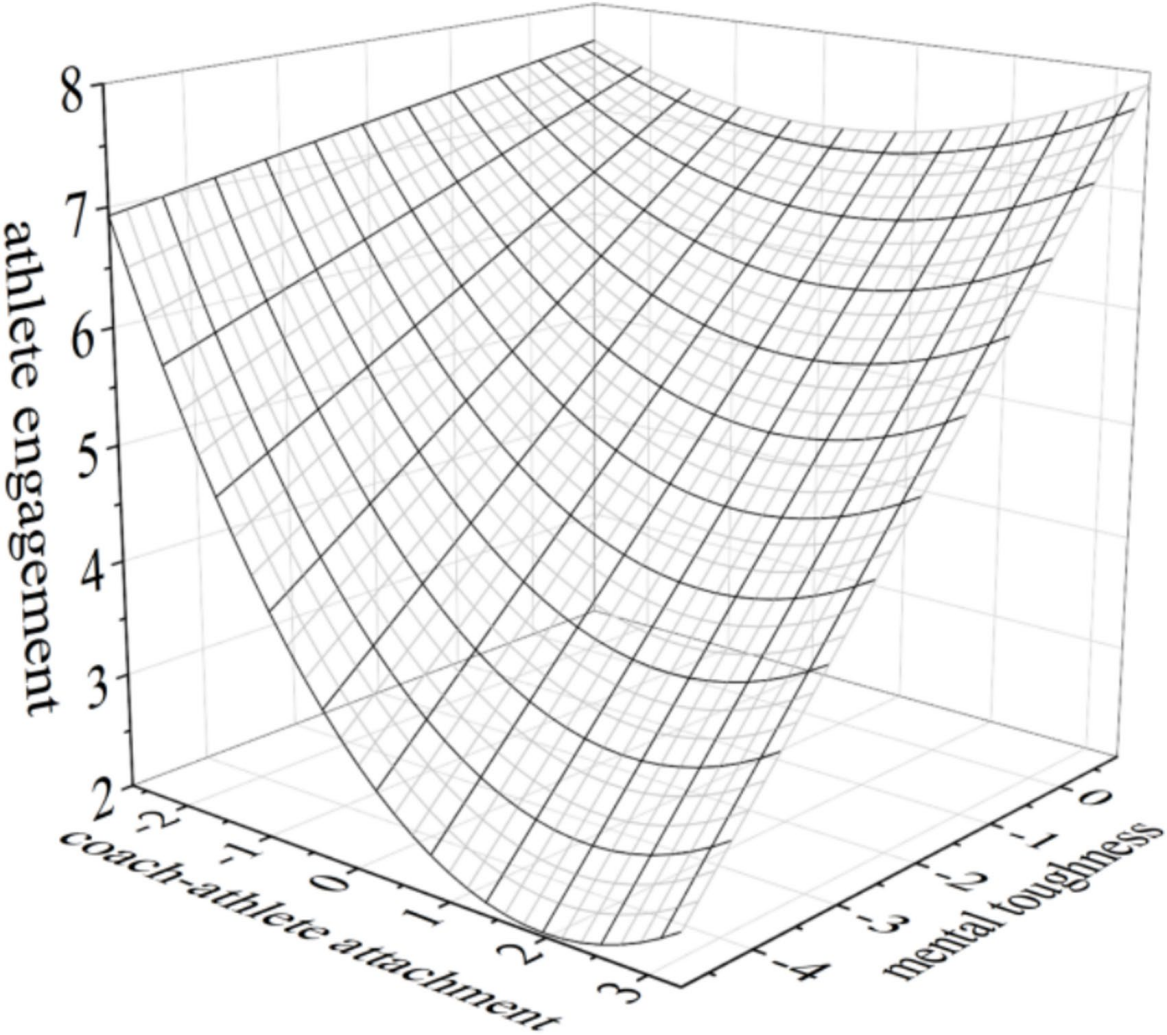
The moderating effect of mental toughness on the U-shaped relationship between coach athlete attachment and athlete engagement. Y (X, Z) = 6.515–0.137X + 0.092×^2^ + 0.157XY-0.028X^2^Y + 0.752Y.Coach athlete attachment∈[−2.684, 3.316]; athlete engagement∈[2, 8]; mental toughness∈[−4.80, 0.86].

Finally, to visually present the moderating effect of mental toughness, three-dimensional response surface plots were generated using Origin2021, as shown in Figures 3, 4.

## Discussion

### The U-shaped effect of coach-athlete attachment on thriving and athlete engagement

Hierarchical regression analysis revealed that the relationship between coach-athlete attachment and both thriving and athlete engagement exhibited a U-shaped pattern of “decline-then-rise.” Furthermore, this U-shaped relationship was characterized by asymmetry, with the left-side path being longer than the right-side path. Specifically, when coach-athlete attachment was at a low level, athletes’ thriving and athlete engagement were at their highest. However, as coach-athlete attachment increased, thriving and athlete engagement initially declined. This suggested that anxious attachment and avoidant attachment negatively predicted thriving and athlete engagement. This finding aligned with prior studies (Davis et al., 2021) and further corroborates that low levels of anxious and avoidant attachment constituted what was widely recognized as “secure attachment” in academia (Baer and Martinez, 2006; Raby et al., 2017). The study further discovered that when coach-athlete attachment reached a moderately high level, thriving and athlete engagement began to increase, indicating that coach-athlete attachment also had a positive facilitative effect on these outcomes. Moderate levels of anxious and avoidant attachment could stimulate higher levels of thriving and athlete engagement among athletes. Coach-athlete attachment represented a unique form of intimate relationship, and such relationships inherently entailed stress (Zhu and Li, 2017), including interaction pressure and responsibility constraints (Qiao, 2023). From the perspective of Conservation of Resources (COR) theory, individuals in stressful environments experienced both the loss and gain of psychological resources, which created two distinct pathways: a loss spiral and a gain spiral (Halbesleben et al., 2014). This dual-path “loss-gain” mechanism underpins the U-shaped relationship between coach-athlete attachment and thriving/athlete engagement.

This finding is significant for deepening the understanding of coach-athlete attachment and its relationship with thriving and athlete engagement. It helps explain inconsistencies in previous research findings and provides empirical evidence and theoretical guidance for coaching practices, particularly for effective communication and interaction with athletes of varying attachment types and levels. Specifically, Coaches should recognize the attachment tendencies of athletes and understand the psychological traits and behavior patterns associated with different attachment styles. Coaches should leverage the positive aspects inherent in both anxious and avoidant attachment styles, adopting differentiated communication and interaction strategies to enhance athlete thriving and engagement. For example, avoidant athletes may appear uncooperative or distant in interactions with coaches, but this often reflects a lack of security and trust, as well as a strong need for independence. Coaches should create a supportive and flexible training environment for these athletes, offering timely care and support to help them build trust and security. In contrast, anxious athletes seek closer relationships with their coaches and often worry excessively about their bonds, showing signs of dependency. Coaches should offer care and constructive feedback, emphasizing their strengths and progress. At the same time, coaches should manage the closeness of the relationship, helping anxious athletes understand their emotional needs, providing balanced emotional support, and fostering self-awareness and independent decision-making to reduce dependency.

### The mediating role of thriving

The analysis results showed that thriving significantly and positively impacted athletes’ athlete engagement, which aligned with previous research findings (Gu et al., 2023). However, this study further revealed that thriving mediated the U-shaped relationship between coach-athlete attachment and athlete engagement. Self-Determination Theory (SDT) is one of the theoretical frameworks for explaining the formation mechanism of athlete engagement (Ye, 2022). According to this theory, social environmental factors influence individuals’ cognition and behavior through autonomous motivation. In this study, thriving represented autonomous motivation, reflecting athletes’ positive, self-directed states during sports training, as well as their experiences of vitality and self-growth. Coach-athlete attachment served as a social environmental factor, embodying athletes’ perceptions of their coaches’ behaviors and attitudes, as well as the quality of the coach-athlete relationship and the nature of their interactions. Athlete engagement, in turn, corresponded to the resulting cognition and behavior of individuals. Specifically, the level and type of coach-athlete attachment resulted from the combined influence of coaches’ behaviors and athletes’ early family experiences. As an external social environmental factor, this attachment could either positively promote or negatively hinder athletes’ thriving. Thriving, as the external manifestation of intrinsic motivation, drove athletes to exhibit vitality and improvement due to their inherent interest in training and competition activities. This intrinsic interest and motivation subsequently altered athletes’ cognition and behaviors toward training, thereby enhancing their level of athlete engagement.

Coaches should take targeted actions to enhance athlete thriving, based on a clear understanding of the factors that predict thriving. Factors such as autonomy in decision-making, ample information sharing, a climate of trust and respect, and performance feedback can stimulate individual thriving (Spreitzer and Porath, 2014). Furthermore, the fulfillment of athletes’ basic psychological needs served as proximal antecedents to thriving, while supportive coaching behaviors and harmonious, close relationships with coaches acted as distal antecedents (May and Luo, 2021). Therefore, coaches can provide systematic support to athletes by focusing on fulfilling their basic psychological needs. For example, in terms of competence support, coaches should provide a conducive training environment, clarify task structures, set realistic goals, offer high-quality and targeted technical and tactical guidance, and provide detailed feedback. In terms of relationship support, coaches should offer genuine respect, understanding, and care for athletes, build a harmonious and close coach-athlete relationship, and foster an open, equal, and trusting team atmosphere. In terms of autonomy support, coaches should delegate decision-making authority, give athletes opportunities for independent decision-making, incorporate their reasonable feedback, and allow athletes to participate in technical analysis and the development of training and competition plans.

### The moderating role of mental toughness

The analysis results of the moderation effect indicated that mental toughness significantly moderated the U-shaped relationship between coach-athlete attachment and thriving, as well as athlete engagement. Specifically, as the level of mental toughness increased, the negative impact of coach-athlete attachment on thriving and athlete engagement diminished, while the positive impact becomes more pronounced. As shown in Figures 3, 4, when mental toughness shifted from low to high, the turning point of the U-shaped curve rose significantly, the curve shifted upward overall, and its opening became smoother. Moreover, the positive gain effect of coach-athlete attachment on thriving and athlete engagement surpassed the depletion effect, and the U-shaped relationship between coach-athlete attachment and thriving nearly disappeared. This phenomenon could be attributed to two key factors. First, athletes with high mental toughness exhibited better emotional regulation and greater cognitive flexibility, which enabled them to manage attachment dynamics with their coaches more effectively. These athletes were also more adept at utilizing various resources to amplify the positive effects of both avoidant and anxious attachment. As a result, the positive impact of coach-athlete attachment on athlete engagement, particularly thriving, was strengthened. Second, athletes with high mental toughness were more likely to exhibit attentional bias toward positive information while avoiding the deliberate processing of negative stimuli. This cognitive style helped them mitigate the negative impact of coach-athlete attachment, allowing them to more effectively leverage the relationship for greater thriving and engagement. In essence, mental toughness played a crucial role in shaping the resource acquisition process by buffering the negative aspects of attachment while enhancing its positive effects. This enabled a more pronounced gain spiral, ultimately fostering greater thriving and athlete engagement. As Pietrzak et al. (2009) and Crust and Swann (2013) noted, mental toughness, as a form of positive psychological capital, not only buffered stress but also produced general gains. Moreover, regression analysis results showed a significant positive correlation between mental toughness and athletes’ thriving (*β* = 0.572) and athlete engagement (*β* = 0.31). These findings aligned with previous studies (e.g., Gucciardi et al., 2017; Ye, 2014). Athletes with higher levels of mental toughness exhibited stronger autonomous motivation, greater confidence, and a stronger sense of commitment, making them more likely to engage in training with high levels of thriving. Evidently, mental toughness was not only an effective remedy for mitigating the negative effects of coach-athlete attachment but also a critical determinant for enhancing thriving and athlete engagement levels.

Therefore, coaches should focus on and strive to cultivate athletes’ mental toughness. The key to enhancing mental toughness lies in creating a certain degree of adversity for athletes, encouraging them to engage in motivational reflection in the face of setbacks (Crust and Clough, 2011). Coaches can help athletes step out of their “comfort zone” by setting up challenging training situations, encouraging them to independently solve various problems encountered in training and competitions, and assigning more challenging training goals and competition tasks. Coaches should guide athletes to reflect on difficulties and failures, helping them transform these experiences into challenges and motivation. It is important to note that athletes with low mental toughness were more susceptible to the negative impact of insecure attachments, such as anxious and avoidant attachment. Therefore, extra attention should be paid to the adverse effects of insecure attachments on athletes with low mental toughness. If conditions allow, psychological skills training, such as breathing exercises, self-talk, mindfulness training, and imagery training, can be implemented for athletes with low mental toughness, as these are effective methods for enhancing their mental toughness (Ajilchi et al., 2019).

## Limitations and future directions

The results of this study have certain practical significance and theoretical value for the construction of secure coach-athlete attachment, the enhancement of athlete engagement, and coaching practices. However, there are some limitations to this study, and future research should explore related issues in greater depth. First, this study mainly uses self-report measures from athletes. To reflect the situation more objectively and comprehensively, future studies could adopt a multi-source evaluation approach, collecting and analyzing data from both coaches and athletes. Second, this study only included athletes from certain team sports. It remains unclear how coach-athlete attachment affects athlete engagement in athletes from other sports, and whether the results and mechanisms differ from those observed in team sports. This issue warrants further investigation in future studies. Third, this study is a cross-sectional quantitative study. Future research could employ qualitative methods to more deeply analyze the specific contexts, mechanisms, and reasons through which coach-athlete attachment influences athlete engagement. Longitudinal studies could also be conducted to clarify the causal relationships between the variables.

## Conclusion

The findings show that coach-athlete attachment and its sub-dimensions (avoidant attachment and anxious attachment) can have a U-shaped effect on thriving, as well as on athlete engagement. Furthermore, there are differences in the trajectory of the U-shaped effect, with the left side of the curve being longer and the right side shorter, forming an asymmetric U-shaped pattern. Thriving significantly positively influences athletes’ athlete engagement and can also act as an instantaneous mediator in the U-shaped relationship between coach-athlete attachment and athlete engagement. Additionally, mental toughness significantly moderates the U-shaped effect of coach-athlete attachment on both thriving and athlete engagement. This study provides valuable insights into the relationships among coach-athlete attachment, thriving, athlete engagement, and mental toughness. It helps explain the inconsistencies in findings from previous related research and offers empirical evidence and theoretical guidance for coaching practices.

## Data Availability

The datasets presented in this study can be found in online repositories. The names of the repository/repositories and accession number(s) can be found in the article/supplementary material.
